# Expression Analysis of Fibronectin Type III Domain-Containing (FNDC) Genes in Inflammatory Bowel Disease and Colorectal Cancer

**DOI:** 10.1155/2019/3784172

**Published:** 2019-04-09

**Authors:** Tilo Wuensch, Jonas Wizenty, Janina Quint, Wolfgang Spitz, Madeleen Bosma, Olaf Becker, Andreas Adler, Wilfried Veltzke-Schlieker, Martin Stockmann, Sascha Weiss, Matthias Biebl, Johann Pratschke, Felix Aigner

**Affiliations:** ^1^Department of Surgery, Campus Charité Mitte and Campus Virchow-Klinikum, Charité-Universitätsmedizin Berlin, Augustenburger Platz 1, 13353 Berlin, Germany; ^2^Medical Department, Division of Hepatology and Gastroenterology (including Metabolic Diseases), Campus Virchow Klinikum, Charité-Universitätsmedizin Berlin, Augustenburger Platz 1, 13353 Berlin, Germany; ^3^Praxis Dr. med. Wolfgang Spitz, Gastroenterologie am Mexikoplatz, Beerenstrasse 50, 14163 Berlin, Germany; ^4^Department of Clinical Chemistry, St. Antonius Hospital, Koekoekslaan 1, 3435 CM Nieuwegein/Utrecht, Netherlands

## Abstract

**Background:**

Fibronectin type III domain-containing (*FNDC*) proteins fulfill manifold functions in tissue development and regulation of cellular metabolism. *FNDC4* was described as anti-inflammatory factor, upregulated in inflammatory bowel disease (IBD). *FNDC* signaling includes direct cell-cell interaction as well as release of bioactive peptides, like shown for *FNDC4* or *FNDC5*. The G-protein-coupled receptor 116 (*GPR116*) was found as a putative FNDC4 receptor. We here aim to comprehensively analyze the mRNA expression of *FNDC1*, *FNDC3A*, *FNDC3B*, *FNDC4*, *FNDC5*, and *GPR116* in nonaffected and affected mucosal samples of patients with IBD or colorectal cancer (CRC).

**Methods:**

Mucosa samples were obtained from 30 patients undergoing diagnostic colonoscopy or from surgical resection of IBD or CRC. Gene expression was determined by quantitative real-time PCR. In addition, *FNDC* expression data from publicly available Gene Expression Omnibus (GEO) data sets (GDS4296, GDS4515, and GDS5232) were analyzed.

**Results:**

Basal mucosal expression revealed higher expression of *FNDC3A* and *FNDC5* in the ileum compared to colonic segments. *FNDC1* and *FNDC4* were significantly upregulated in IBD. None of the investigated *FNDCs* was differentially expressed in CRC, just *FNDC3A* trended to be upregulated. The GEO data set analysis revealed significantly downregulated *FNDC4* and upregulated *GPR116* in microsatellite unstable (MSI) CRCs. The expression of *FNDCs* and *GPR116* was independent of age and sex.

**Conclusions:**

*FNDC1* and *FNDC4* may play a relevant role in the pathobiology of IBD, but none of the investigated *FNDCs* is regulated in CRC. GPR116 may be upregulated in advanced or MSI CRC. Further studies should validate the altered *FNDC* expression results on protein levels and examine the corresponding functional consequences.

## 1. Introduction

Fibronectin type III domain-containing 4 (*FNDC4*) was recently found by Bosma et al. as a novel anti-inflammatory factor, upregulated in human and murine intestinal inflammation and with therapeutic potential in inflammatory bowel disease (IBD) [1]. *FNDC4* is one of in total eight so far identified members of the FNDC protein family in humans [2]. The FNDC proteins are characterized by at least one fibronectin type III domain (FN3). Their various functions include tissue development and cell adhesion, migration, and proliferation. Several studies showed that *FNDCs* are regulated by microRNAs [3–5]. Additionally, other mechanisms such as *FNDC4* regulation by *TGF-β* and corticoid receptor involvement for *FNDC5* expression were also described [1, 6]. General expression analyses and functional reports exist for *FNDC1*, *FNDC3A*, *FNDC3B*, *FNDC4*, and *FNDC5*. *FNDC1* activates G-protein signaling 8 (*AGS8*) and was previous described as a regulator of cardiovascular functions. In particular, *FNDC1* plays a role in *VEGF*-mediated angiogenesis and is required for hypoxia-induced apoptosis in cardiomyocytes [7, 8]. Quantitative trait loci (QTL) analysis found protein-altering mutations in *FNDC1* that are associated with arterial hypertension [9]. On the other hand, *FNDC1* expression correlates with aggressive prostate cancer [3]. *FNDC3A* contributes to the synthesis of extracellular matrix in odontoblasts and to spermatogenesis [10, 11]. It interacts with the human leukocyte antigen *DRB1* in the pathogenesis of rheumatoid arthritis and expression analyses in colonic tissue showed that *FNDC3A* is upregulated in sporadic CRC [12, 13]. *FNDC3B* promotes epithelial-to-mesenchymal transition and activates multiple cancer pathways, for example, in squamous cell carcinoma, acute promyelocytic leukemia, and hepatocellular carcinoma [14–17]. To date, *FNDC5* is the most extensively studied *FNDC*, mainly because it is the launch vehicle of the peptide hormone irisin, which was proposed to facilitate the conversion of white adipose tissue into beige adipose tissue [18]. Yet, this concept is not completely understood and controversially discussed [19]. These mainly recently published data on *FNDCs* reveal a variety of functions in healthy and diseased conditions in multiple organs and testify upcoming interest in structured analysis of these proteins.

Similar to *FNDC5*, *FNDC4* is cleaved and releases a functional active peptide [1]. The exact receptor or binding partner to facilitate downstream signaling is, so far, not fully understood. However, Georgiadi et al. revealed in an *in vitro* binding study the orphan G-protein-coupled receptor 116 (*GPR116*) as a putative functional FNDC4 receptor candidate [20]. *GPR116* is expressed in various tissues, including lung, kidney, or fat, and it has been described to be overexpressed in metastatic colorectal cancer (CRC) or breast cancer [21, 22].

The expression of *FNDCs* and *GPR116* in human IBD and CRC has not been investigated orderly. Therefore, this study is aimed at exploring the expression of *FNDC1*, *FNDC3A*, *FNDC3B*, *FNDC4*, *FNDC5*, and *GPR116* in nonaffected and affected mucosa samples of patients with IBD or CRC. Information about the regulation of the basal gene expression could be helpful for a better understanding of pathophysiological disease mechanisms and for the identification of potential therapeutic targets.

## 2. Materials and Methods

### 2.1. Human Sample

IBD samples were collected at the Medical Department, Division of Hepatology and Gastroenterology, Campus Virchow-Klinikum at Charité–Universitätsmedizin Berlin, or at Praxis Dr. med. Wolfgang Spitz from patients with ulcerative colitis (UC) or Crohn's disease (CD) undergoing routine diagnostic colonoscopy. CRC samples were collected at the Department of Surgery at Charité–Universitätsmedizin Berlin from patients undergoing surgical resection. Samples that showed no pathological signs macroscopically or histologically were included as controls (nonaffected samples). Collected samples were immediately frozen in liquid nitrogen and stored at -80° until further use. The study was approved by the local ethics committee (registry number EA2/021/16) and conducted in accordance with the Declaration of Helsinki. Written informed consent was obtained from each patient before enrolment.

### 2.2. Patients' Characteristics

In this study, 20 IBD patients, 10 UC patients, and 10 CD patients participated. In total, 44 double-bite mucosal biopsies (two bites per pass of the forceps [23]) covering the proximal-distal axis were obtained by experienced gastroenterologists. Samples were allocated histologically into three groups: IBD nonaffected, UC affected, and CD affected (Table 1). Patients' clinical disease activity was assessed using two common scores: the Mayo Score for ulcerative colitis and the Harvey-Bradshaw Index for Crohn's disease. The Mayo Score was described by Schroeder et al. [24] in 1987 and composites four categories, including stool frequency, rectal bleeding, mucosal appearance at endoscopy, and physician rating of disease activity, with a maximum score of 12. The Harvey-Bradshaw Index was introduced in 1980 as a simpler version of the Crohn's disease activity index (CDAI) containing only clinical parameters, including general well-being, abdominal pain, number of liquid stools per day, abdominal mass, and complications [25]. The sum of items categorizes the patients into remission (<5), mild disease (5-7), moderate disease (8-16), and severe disease (>16). Additionally, the current medication and basic laboratory findings of study patients were recorded (Table 1). The 8 patients in the UC affected group included 7 males and 1 female, with a median age of 39 (26-54) years. These patients were most diagnosed with pancolitis (5/8) and a mild disease activity (7/8) according to the Mayo Score. The 8 patients in the CD affected group included 3 males and 5 females, with a median age of 32 (28-60) years. 50% of these patients were in clinical remission according to the Harvey-Bradshaw Index; they mainly displayed ileocolonic disease activity. At the time of enrolment, all patients received anti-inflammatory medication.

In addition, 10 patients with CRC enrolled in this study; one of those patients participated with two synchronous CRCs. Expression in affected samples was compared with nonaffected samples taken from adjacent mucosa of each patient. All CRC patients were men and their median age was 64.5 years. 6/11 carcinoma samples were originated in the rectum. 8/10 patients did not receive any neoadjuvant therapy. CRCs were mainly staged as UICC stage I or II and histologically moderate differentiated (G2). 8/11 CRC were microsatellite stable; two pathological analyses did not reveal information about microsatellite stability. Clinical data, histological analysis, and laboratory findings of CRC subjects are listed in Table 2.

### 2.3. Bioinformatic Analysis of Gene Expression Profiles

Data sets containing array-based gene expression profile data (normalized expression values) were retrieved from the Gene Expression Omnibus (GEO) platform. The normalized expression values (given in arbitrary units) were analyzed for differential gene expression of *FNDCs* and *GPR116* in CRC cell lines (GDS4296) and microsatellite unstable carcinoma (MSI CRCs) (GDS4515) as well as in early- vs. late-onset carcinoma in males and female (GDS5232), using one-way ANOVA for multiple comparison test and *t*-tests for comparing two groups.

### 2.4. Quantitative Real-Time RT-PCR

RNA was extracted using the PureLink™ RNA Mini Kit (Invitrogen, CA, USA) according to manufacturer's protocol using a rotating homogenizer (Retsch MM400, Haan, Germany). Quality and quantity controls were performed for each extract using the NanoDrop™ 2000 Spectrophotometer (Thermo Scientific, DE, USA). Immediately after RNA extraction, cDNA synthesis was performed using the High-Capacity cDNA Reverse Transcription Kit (Applied Biosystems, CA, USA) following manufacturer's instructions. The cDNA was stored at -20° until use. For qRT-PCR reactions, the GoTaq® qPCR Master Mix (Promega, WI, USA) was used and a 7500 Real-Time PCR Cycler System (Applied Biosystems, CA, USA) to run the PCRs. Primer sequences are listed in Table 3. The inflammatory markers chemokine (C-C motif) ligand 2 *(CCL2)*, interleukin 4 *(IL-4)*, and tumor necrosis factor *(TNF)* have been shown to be upregulated in mucosal biopsies of patients with inflammatory bowel disease [26–28] and were therefore measured additionally to validate inflammatory processes in our mucosa samples. Cycling conditions were as follows: initial denaturation for 10 min at 95°C, followed by 40 cycles of denaturation at 95°C for 15 s and annealing/extension at 60°C for 60 s. Melt curve analysis was carried out from 60 to 95°C with a temperature transition rate of 0.1°C/s. The expression of *FNDCs* and inflammatory markers was, if not otherwise indicated, normalized to the housekeeping genes actin-beta (*ACTB*) and TATA box-binding protein (*TBP*) and expressed as fold change to the expression in nonaffected samples, using the 2^−ΔΔCt^ equation [29]. Amplified PCR product lengths were qualitatively analyzed by electrophoresis on 3% agarose gels containing ethidium bromide to determine predicted product length.

### 2.5. Statistical Analysis

All statistical analyses were performed using the Statistical Package for the Social Sciences (SPSS, version 25.0, IBM, NY, USA) or GraphPad Prism 5.0 (GraphPad Software, CA, USA). The results are expressed as mean ± SEM. Statistical comparisons between two groups were performed using *t*-tests. Grubbs' test was performed to identify outliers. Statistical significant differences were defined as *p* < 0.05(^∗^), *p* < 0.01(^∗∗^), and *p* < 0.001(^∗∗∗^).

## 3. Results

### 3.1. Gene Expression of *FNDCs* and *GPR116* in Nonaffected Mucosal Samples along the Proximal-Distal Axis

**Figure 1:**
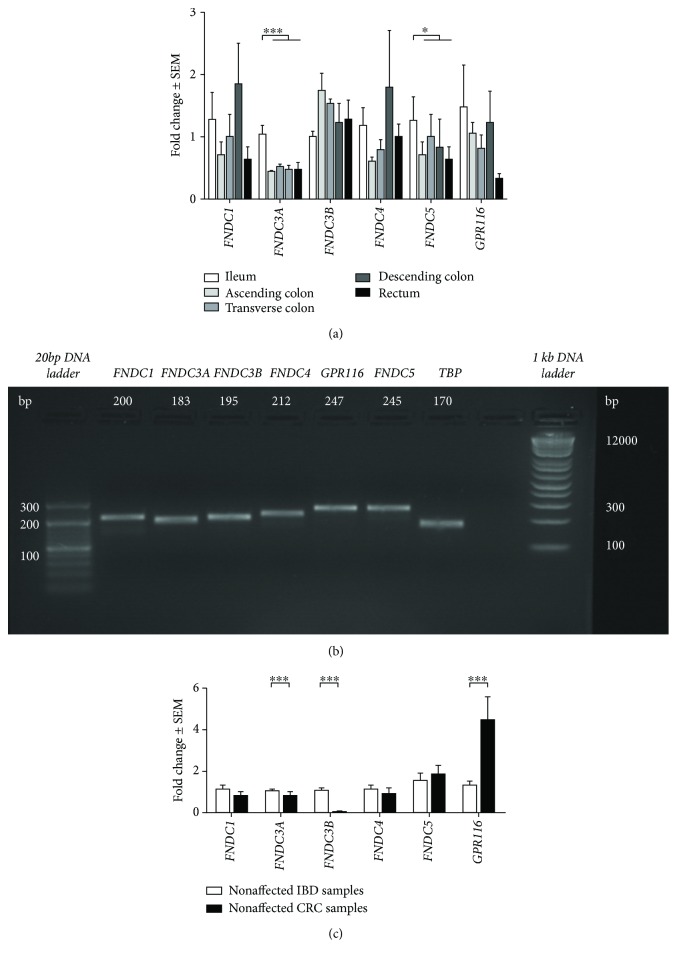
*FNDCs* and *GPR116* expression in nonaffected human mucosal samples. *FNDC1*, *FNDC3A*, *FNDC3B*, *FNDC4*, *FNDC5*, and *GPR116* in nonaffected mucosal samples along the proximal-distal axis (*n* = 4-5) (a). Representative agarose gels of qRT-PCR products, including the expected amplicon lengths for each gene (b). Quantification of *FNDC1*, *FNDC3A*, *FNDC3B*, *FNDC4*, *FNDC5*, and *GPR116* in nonaffected samples of patients with IBD (*n* = 22) and CRC (*n* = 7-10) (c). Data are expressed relative to the expression in nonaffected IBD samples and normalized to the housekeeping genes *ACTB* and *TBP*. ^∗^ *p* < 0.05, ^∗∗^ *p* < 0.01, and ^∗∗∗^ *p* < 0.001.

Basal *FNDC* expression was assessed in nonaffected samples along the proximal-distal axis starting from the ileum to the rectum (Figure 1(a)). As all samples in this analysis were nonaffected, the expression was calculated in fold change to expression of the ileum. Significant expression differences in nonaffected mucosal samples along the proximal-distal axis were found for *FNDC3A* and *FNDC5*; both were expressed at a higher level in the ileum compared to colonic segments (*p* < 0.0001 for *FNDC3A*, *p* = 0.0118 for *FNDC5*). No statistical expression differences along the proximal-distal axis were found for *FNDC1*, *FNDC3B*, *FNDC4*, and *GPR116*, respectively. For qRT-PCR result verification, the actual amplicon sizes were determined by ethidium bromide gel electrophoresis and compared to predicted lengths (Figure 1(b)).

### 3.2. Gene Expression of *FNDCs* and *GPR116* in Nonaffected Mucosal Samples of IBD vs. CRC Patients

No differences were found for the expression of *FNDC1* (*p* = 0.1760), *FNDC4* (*p* = 0.4374), or *FNDC5* (*p* = 0.6781) in nonaffected mucosal samples of IBD compared to the expression in nonaffected mucosal samples of CRC patients, respectively (Figure 1(c)). Significantly higher levels of *FNDC3A* and *FNDC3B* were found in nonaffected samples of IBD patients, as compared to nonaffected samples of CRC patients. *GPR116* was expressed at higher levels in CRC samples, as compared to nonaffected samples of IBD patients.

### 3.3. *FNDC1* and *FNDC4* Are Significantly Upregulated in Inflamed Colonic Mucosal Samples

**Figure 2:**
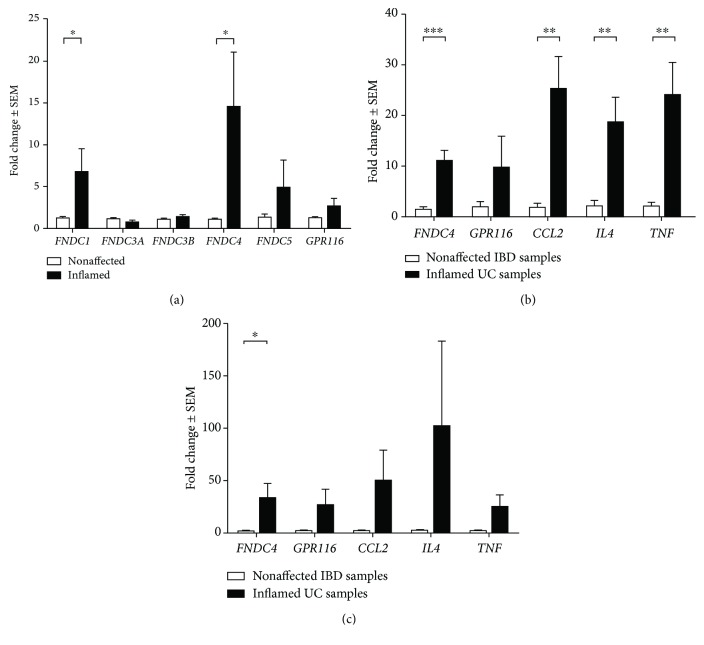
*FNDCs* and *GPR116* expression in nonaffected vs. inflamed mucosal samples of IBD patients. Expression levels of *FNDC1* and *FNDC4* were significantly higher in inflamed samples (*n* = 14) than in nonaffected samples (*n* = 22) of IBD patients (a). *FNDC4*, *GPR116*, *CCL2*, *IL-4*, and *TNF* expression levels in samples of active ulcerative colitis (UC, *n* = 8) (b) or Crohn's disease (CD, *n* = 8) (c), as compared to nonaffected samples. ^∗^ *p* < 0.05, ^∗∗^ *p* < 0.01, and ^∗∗∗^ *p* < 0.001.

Mucosal samples of IBD patients were collected from actively inflamed and nonaffected sites. In macroscopically and histologically inflamed samples, *FNDC1* and *FNDC4* were significantly upregulated, compared to all nonaffected samples used previously for Figure 1(a) (*FNDC1* 5.5-fold, *p* = 0.0244; *FNDC4* 13.8-fold, *p* = 0.0149). *FNDC3A*, *FNDC3B*, *FNDC5*, and *GPR116* showed no differences between inflamed and nonaffected samples (Figure 2(a)). Focusing on the gene expression of *FNDC4* and *GPR116*, *FNDC4* is upregulated in active UC (7-fold increase, *p* < 0.001) as well as in active CD (22-fold increase, *p* = 0.0462) (Figures 2(b) and 2(c)). A more pronounced inflammatory reaction was observed in UC samples, as reflected by the expressions of *CCL2*, *IL-4*, and *TNF*, that were only significantly regulated in UC samples (*p* < 0.01) and not in CD samples (Figures 2(b) and 2(c)). *GPR116* was not specifically regulated in UC or CD (*p* = 0.192 in UC, *p* = 0.1835 in CD), although it tended to increase.

### 3.4. Unchanged *FNDCs* and *GPR116* Expression in CRC

**Figure 3:**
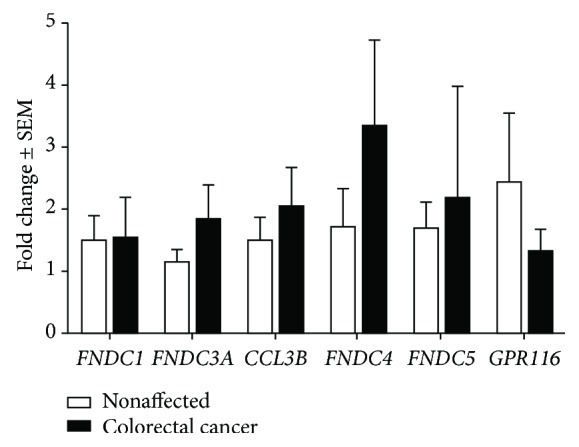
*FNDCs* and *GPR116* expression in colorectal cancer. No significant expression differences were found in a paired-sample *t*-test analysis for any of the investigated genes between cancerous samples and the surrounding nonaffected samples (*n* = 8-10).

CRC samples were compared to surrounding nonaffected samples that were previously used in the calculation for Figure 1(c) (*n* = 8-10); however, none of the investigated genes was differentially expressed (Figure 3). *FNDC3A* showed the strongest tendency of increasing expression in CRC, yet not reaching statistical significance (*p* = 0.097).

### 3.5. *FNDCs* and *GPR116* Expression in GEO Data Sets

**Figure 4:**
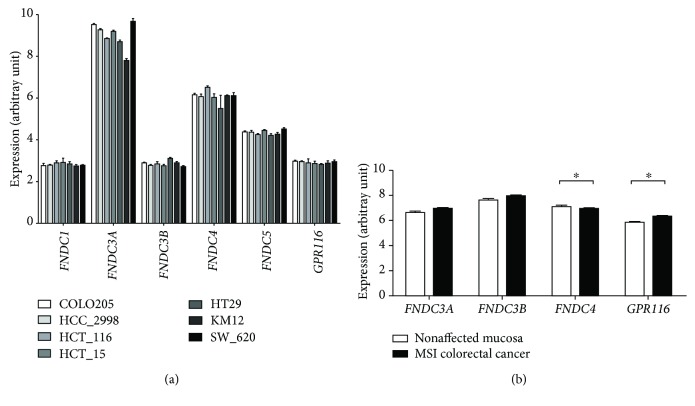
Microarray expression values retrieved from the GEO database (data sets GDS4296 and GDS4515). *FNDCs* and *GPR116* are expressed in several colorectal cancer cell lines from the NCI-60 panel (24) (a). In human MSI colorectal cancer, *FNDC4* is significantly downregulated (*p* = 0.047), whereas *GPR116* is upregulated (*p* = 0.014) (25) (b). ^∗^ *p* < 0.05.

Publicly available data sets of the GEO database were analyzed to complement our findings with previous comparable studies. First, microarray expression of *FNDCs* in seven human colonic tumor cell lines from the NCI-60 panel were obtained (GDS4296) [30]. All *FNDCs* were expressed, whereas *FNDC3A* displayed the highest expression values (mean 2.4-fold higher) in all cell lines (Figure 4(a)). Significant expression differences (*p* < 0.05) in analyzed cell lines were found regarding *FNDC3A* and *FNDC3B* (significance bars are not shown for overview purposes). In the microsatellite-unstable colorectal cancer data set (GDS4515) of human MSI CRC samples (*n* = 34), we analyzed the gene expressions compared to nonaffected colonic mucosa (*n* = 17) [31]. The expression data of *FNDC3A*, *FNDC3B*, *FNDC4*, and *GPR116* were available and showed a small but significant downregulation of *FNDC4* in MSI CRCs (*p* = 0.047, Figure 4(b)), while *FNDC3A* (*p* = 0.055) and *FNDC3B* (*p* = 0.127) remained unchanged. *GPR116* was significantly upregulated (*p* = 0.014). To test for possible confounding by age or sex, we analyzed expression data in early- and late-onset CRCs in females (*n* = 21) and males (*n* = 25), diagnosed at an age of 28-53 years (*n* = 27) or 69-87 years (*n* = 19) (GDS5232) [32]. Data were available for *FNDC3A*, *FNDC4*, and *GPR116*, which showed neither age- (*p* = 0.548, *p* = 0.906, and *p* = 0.160) nor sex-dependent differences (*p* = 0.438, *p* = 0.629, and *p* = 0.547).

## 4. Discussion

In this exploratory study, we investigated the gene expression of *FNDC1*, *FNDC3A*, *FNDC3B*, *FNDC4*, *FNDC5*, and *GPR116* in IBD and CRC. Our main mRNA expression results as well as the proposed gene function are listed in Table 4. First, basal gene expression was analyzed in the proximal-distal row showing similar expressions for FNDC1, FNDC3B, FNDC4, and GPR116, whereas FNDC3A and FNDC5 were dominantly expressed in the ileum. The unchanged expression for FNDC4 has been also previously reported [1]. The biological relevance of variable regional expression pattern still remains unknown.

Concerning IBD, it was reported that FNDC4 is upregulated in the mucosa and acts as an anti-inflammatory factor via the downregulation of proinflammatory genes and phagocytosis [1]. Our data confirmed the upregulation of FNDC4 in UC and CD samples. Moreover, we found FNDC1, as a second gene of the FNDC family, upregulated in IBD. This might suggest a functional role of FNDC1 in IBD, which was until now foremost known for its pathological role in cardiocirculatory disorders and prostate cancer [3, 7–9]. The upregulation of FNDC1 and FNDC4 expression and the subsequent translation might affect intracellular or cell-cell signaling. So far, several FNDC receptor candidates have been described in different organs or cells. It has been described that FNDC1 regulates the androgen receptor in prostate cancer [3]. Furthermore, FNDC1 forms complexes with G protein beta gamma subunit and connexins in cardiomyocytes and regulates several VEGF receptors in endothelial cells [7, 8, 33]. FNDC4 has been shown to suppress inflammation via AMP-activated protein kinase (AMPK) phosphorylation and heme oxygenase-1 (HO-1) expression in adipocytes [34]. It has also been shown to inhibit osteoclast formation via the suppression of NF-*κ*B and downregulation of CXCL10 [35]. Another FNDC4 receptor candidate was supposed by Georgiadi et al., who found that FNDC4 binds to GPR116 [20]. In our analysis, GPR116 was not significantly regulated in IBD samples. All our attempts to establish the detection of FNDC4 protein in mucosal samples failed, as available antibodies were unable to provide a specific staining and were distorted by autofluorescence of IBD samples (Supplemental figure (available here)). We discussed this phenomenon earlier in a separate publication [36]. Therefore, also the exact cellular localization of FNDC4 protein during inflammatory activity remains unknown. One possible mechanism might involve the activation of macrophages [1]. Physiological functions of *FNDC5*, a close paralog of *FNDC4*, include effects on metabolism, e.g., improved glucose metabolism by upregulating uncoupling protein-1 (UCP1) via phosphorylation of the p38 mitogen-activated protein kinase (p38 MAPK), cardiovascular function, e.g., reduced atherosclerosis, skeletal muscle, e.g., myogenesis via NO-dependent mechanisms, and central nervous system, e.g., increased hippocampal neurogenesis [37]. Our data confirms the expression profiles of *FNDC5* in IBD, as shown by Bosma et al., who found *FNDC5* either downregulated or unchanged in several mouse models of inflammation [1].

Concerning CRC, neither *FNDCs* nor *GPR116* was significantly regulated in our analysis. So far, no information about *FNDC1* and *FNDC4* expression in CRC were available. As we found no significant changes at the transcription level, our results suggest that *FNDC1* and *FNDC4* are not relevant factors in intestinal dysplasia, although we cannot exclude a role for (post)translational regulation of *FNDCs*. In the microsatellite-unstable colorectal cancer data set, *FNDC4* was small but significantly downregulated. That stays in line with a recent Affymetrix microarray analysis, which showed a downregulation of *FNDC4* in a murine metastatic colon adenocarcinoma cell line [31, 38]. These data might point to a suppressed transcription of *FNDC4* in the described CRC pathologies, although the biological relevance of these small expression differences remains questionable. For *GPR116*, we did not find any expression differences between nonaffected mucosa samples and CRC samples, although it was significantly highly expressed in cancer adjacent mucosa compared to nonaffected mucosa of IBD patients. Yang et al. found a correlation between highly expressed *GPR116* and poor survival outcome in CRC [21]. In their studies, high *GPR116* expression, semiquantitatively graded by immunohistochemical staining, was found in approximately 50% of their patients. However, their study population differed from ours. Yang et al. enrolled 47% stage I-II and 53% stage III-IV CRCs. 38% of CRCs revealed a poor histological differentiation, which correlated with high GPR116 expression. In well/moderate-differentiated CRCs, the *GPR116* expression was foremost low. We showed low *GPR116* expressions in our predominantly moderate differentiated CRCs, which is in line with Yang and colleagues. No information about microsatellite stability were given by Yang et al., nor were rectal cancers analyzed. Our analysis of GEO data sets revealed an upregulated expression of *GPR116* in MSI CRC. MSI is a molecular characteristic in hereditary and sporadic CRCs that features an antitumoral immune response and involves good prognosis [39]. Today, CRCs can be classified into heterogeneous subtypes, e.g., resulting from specific genetic mutations, and therefore, expression and biomarker studies should provide separate analysis in subgroups, e.g., MSI CRC. Yet, our data does not allow any conclusion about a functional relationship between any of the *FNDCs* with *GPR116*. This has to be investigated in further studies.

One study showed increased *FNDC5* immunoreactivity in several gastrointestinal tract cancers including CRC [40]. These findings could not be reproduced in our study. One main methodical difference between the studies is the *FNDC5* analysis on the protein level, while we analyzed mRNA expression. Due to the common lack of congruency between RNA expression and protein abundance, the effects seen on the mRNA levels do not necessarily translate into protein abundance [41].

IBD patients are at high risk to develop CRC in the course of the disease, caused by the continuous tissue inflammation, repair, and remodeling. The CRC risk rises up to 2.4-fold for patients with UC and up to 1.9-fold for patients with CD [42, 43]. Therefore, *FNDC* expression in the course of CRC development based on IBD could help to better understand their importance during the disease progression. However, in this study, only patients with sporadic CRC participated. Just little is known about the role of *FNDCs* in the pathogenesis of CRC, especially if it is based on IBD. Shivakumar et al. analyzed the copy number variations of *FNDC3A* and found it highly amplified in sporadic CRC but not in UC-associated neoplasia [13]. In contrast, positive immunostaining for *FNDC3A* was found in patients with an extensive course of UC, especially with neoplastic changes. In our CRC samples, *FNDC3A* was close to significance (*p* = 0.097) for higher expression in CRC compared to nonaffected surrounding mucosa. That might have been limited due to the relatively low sample number. Further studies should solidify the expression of *FNDC3A* as well as its functional implications in CRC on larger study cohorts comparing homogenous pathologies. *FNDC3B*, the second member of the *FNDC3* subfamily, shares 50% amino acid identity with FNDC3A. It is known that *FNDC3B* is highly amplified in esophageal, lung, ovarian, and breast cancer and it has been associated with the activation of several cancer pathways including PI3-kinase/Akt, Rb1, and TGF-*β* signaling [14]. Recently, Chen et al. propose *FNDC3B* as potential biomarker for lymph node metastatic CRC [44]. Unfortunately, we could not confirm this, since 8/11 of our CRC specimen were lymph node negative. We found *FNDC3B* expression unchanged in IBD and CRC.

## 5. Conclusions

This exploratory gene expression study provides first insights of fibronectin type III domain-containing proteins in IBD and CRC. *FNDC1* and *FNDC4* are upregulated in IBD, while no significant changes for *FNDCs* or *GPR116* were found in CRC. The diagnostic potential and pathomechanistic contribution of *FNDCs* are most likely to be limited to IBD but not CRC development. Still, *GPR116* might be upregulated in advanced or MSI CRC. Further research is required to validate expression differences on the protein level and to elucidate functional consequences, including FNDC receptor interactions as well as signaling mechanisms on the molecular level.

## Figures and Tables

**Table 1:** IBD patients' characteristics.

	IBD nonaffected	UC affected	CD affected
Number of subjects	11 (4 CU, 7 CD)	8	8
Age, median years (range)	45 (27-60)	39 (26-54)	32 (28-60)
Age at first diagnosis (mean ± SD)	30.8 ± 7.2	29.4 ± 6.5	27.5 ± 6.9
Sex (male/female)	6/5	7/1	3/5
Localization			
Ileum	5	0	4
Ascending colon	5	1	0
Transverse colon	6	4	1
Descending colon	5	1	1
Sigmoid colon	1	3	1
Rectum	4	1	1
Mayo Score			
No activity (0-1)	4	0	NA
Mild disease (2-5)	0	7	NA
Moderate/severe disease (≥6)	0	1	NA
Harvey-Bradshaw Index			
Remission (<5)	4	NA	4
Mild disease (5-7)	3	NA	3
Moderate disease (8-16)	0	NA	1
Severe disease (>16)	0	NA	0
Montreal classification			
E1, E2, E3	0/1/3	0/3/5	NA
A1, A2, A3	0/6/1	NA	0/7/1
L1, L2, L3, L4	1/3/3/0	NA	1/4/3/0
B1, B2, B3, B3p	1/4/1/1	NA	1/4/1/2
Medication			
No	0	0	0
5-ASA	2	5	1
Corticosteroids	2	2	2
Immunosuppressives	2	2	0
Biologicals	9	3	6
Small molecule drugs	0	0	1
As combination	4	3	2
Laboratory findings (mean ± SD)			
Erythrocytes (10^12^/L)	4.5 ± 0.46	4.7 ± 0.64	4.6 ± 0.38
Leukocytes (10^9^/L)	8.56 ± 2.82	7.68 ± 2.39	9.61 ± 3.87
Thrombocytes (10^9^/L)	292.8 ± 123	314.7 ± 99.1	344.9 ± 81.9
Hemoglobin (g/dL)	13.3 ± 0.7	12.8 ± 2	13.5 ± 1
CRP (mg/L)	2.1 ± 3.8	7.5 ± 9.9	6.5 ± 9.9
Sodium (mmol/L)	141.2 ± 2.1	140.9 ± 1.2	140.3 ± 2.3
Potassium (mmol/L)	4.01 ± 0.2	4.14 ± 0.3	3.97 ± 0.14

Each column lists characteristics for all patients contributing with samples to the corresponding sample group. Abbreviations: E1: ulcerative proctitis; E2: left-sided UC; E3: pancolitis; A1: below 16 y; A2: between 17 and 40 y; A3: above 40 y; L1: ileal; L2: colonic; L3: ileocolonic; L4: isolated upper disease; B1: nonstricturing, nonpenetrating; B2: stricturing; B3: penetrating; B3p: perianal disease; 5-ASA: 5-aminosalicylic acid; CRP: C-reactive protein.

**Table 2:** CRC patients' characteristics.

Number of subjects	10
Age, median years (range)	64.5 (50-80)
Age at first diagnosis (mean ± SD)	62.3 ± 9.9
Sex (male/female)	10/0
Localization	
Ileum	0
Caecum	0
Ascending colon	1
Transverse colon	1
Descending colon	1
Sigmoid colon	2
Rectum	6
Neoadjuvant therapy	
None	8
Grade 1 (Dworak)	1
Grade 2 (Dworak)	1
UICC stage	
NA	1
Stage I	4
Stage II	3
Stage III	1
Stage IV	1
Histological differentiation	
NA	1
G1	0
G2	8
G3	1
G4	0
Microsatellite stability	
NA	2
MSS	8
MSI	0
KRAS mutation	1
BRAF mutation	1
Positive family history	5
Smokers (pack-years, range)	7 (28, 10-75)
Daily alcohol	5
Laboratory findings (mean ± SD)	
Erythrocytes (10^12^/L)	4.7 ± 0.46
Leukocytes (10^9^/L)	8.21 ± 3.76
Thrombocytes (10^9^/L)	251.1 ± 94.6
Hemoglobin (g/dL)	13.4 ± 2.1
Sodium (mmol/L)	139.6 ± 4.3
Potassium (mmol/L)	4.36 ± 0.55
CEA (*μ*g/L)	7.9 ± 16.7
CA 19-9 (U/mL)	180.5 ± 392.4

The column lists characteristics for all patients with CRC. Abbreviations: UICC: Union for International Cancer Control; NA: not available; G1: well differentiated; G2: moderately differentiated; G3: poorly differentiated; G4: undifferentiated; CEA: carcinoembryonic antigen; CA 19-9: carbohydrate antigen 19-9.

**Table 3:** Primer sequences used for qRT-PCR (5′-3′).

Gene product	Gene accession number	Forward primer sequence	Reverse primer sequence	Product size (bp)
*FNDC1*	NM_032532	GAG CCT TCG ACC ACT GCT AC	ATT TCA TCC AGG CTG TCC AC	200
*FNDC3A*	NM_001079673	GCA CAA GTG AAT TGG GAG GT	CAC AAC ACT CAG AGC CTG GA	183
*FNDC3B*	NM_022763	AGT CTC CCT GTT CGC ACA CT	CTC TGG GCC ATG GTA CAC TT	195
*FNDC4*	NM_022823	GCA CTT CCG AAC TCT CAA GG	TGG GAT TGT TGT TGG AGT CA	212
*FNDC5*	NM_153756	CAT CTC CCA GCA GAA GAA GG	GGT TCC TCC CCA TCT CTT TC	245
*GPR116*	NM_015234	GGA AGC TGT GGT GTG GAA AT	CAG GCG ATA GAA CAG CAT GA	247
*CCL2*	NM_002982	CCC CAG TCA CCT GCT GTT AT	TGG AAT CCT GAA CCC ACT TC	171
*IL-4*	NM_000589	TGA ACA GCC TCA CAG AGC AG	TCA GGA ATC GGA TCA GCT GC	210
*TNF*	NM_000594	CAG AGG GCC TGT ACC TCA TC	GGA AGA CCC CTC CCA GAT AG	219
*ACTB*	NM_001101	GGA CTT CGA GCA AGA GAT GG	AGC ACT GTG TTG GCG TAC AG	234
*TBP*	NM_003194	TAT AAT CCC AAG CGG TTT GC	GCT GGA AAA CCC AAC TTC TG	170

**Table 4:** Summary of IBD and CRC analysis and main proposed functions of *FNDCs* and *GPR116*.

Gene	Regulation in IBD	Regulation in CRC	Proposed function
*FNDC1*	Upregulated	Unchanged	Angiogenesis, apoptosis
*FNDC3A*	Unchanged	Trends upregulated	Organogenesis
*FNDC3B*	Unchanged	Unchanged	Cancer pathway activator
*FNDC4*	Upregulated	Unchanged/downregulated^∗^	Anti-inflammatory
*FNDC5*	Unchanged	Unchanged	Energy metabolism
*GPR116*	Unchanged	Unchanged/upregulated^∗^	*FNDC4* receptor candidate

^∗^depending on CRC subtypes.

## Data Availability

All data used to support the findings of this study are available from the corresponding author upon request.

## Supplementary Materials

Supplementary Materials Supplemental figure: immunofluorescence staining and autofluorescence of inflamed intestinal tissue. (a) A subepithelial cellular IF label is visible with the use of 1st antibody: Anti-FNDC4 (HPA015804, dilution 1 : 50, Sigma-Aldrich) and 2nd antibody: Goat Anti-Rabbit IgG H&L (Alexa Fluor® 594) (ab150080, dilution 1 : 200, Abcam). Colonic crypts are labeled by Cadherin-17 antibody (MAB1032, R&D Systems) with 2nd antibody Goat Anti-Mouse IgG FITC (F0257, dilution 1 : 250, Sigma-Aldrich). DAPI was used for counterstaining. (b) The subepithelial cellular label also appears in a labeling control without antibodies after an excitation with 594 nm and is therefore autofluorescence.
