# Erratum to “Expression Analysis of Fibronectin Type III Domain-Containing (FNDC) Genes in Inflammatory Bowel Disease and Colorectal Cancer”

**DOI:** 10.1155/2020/8691904

**Published:** 2020-06-26

**Authors:** Tilo Wuensch, Jonas Wizenty, Janina Quint, Wolfgang Spitz, Madeleen Bosma, Olaf Becker, Andreas Adler, Wilfried Veltzke-Schlieker, Martin Stockmann, Sascha Weiss, Matthias Biebl, Johann Pratschke, Felix Aigner

**Affiliations:** ^1^Department of Surgery, Campus Charité Mitte and Campus Virchow-Klinikum, Charité-Universitätsmedizin Berlin, Augustenburger Platz 1, 13353 Berlin, Germany; ^2^Medical Department, Division of Hepatology and Gastroenterology (Including Metabolic Diseases), Campus Virchow Klinikum, Charité-Universitätsmedizin Berlin, Augustenburger Platz 1, 13353 Berlin, Germany; ^3^Praxis Dr. Med. Wolfgang Spitz, Gastroenterologie am Mexikoplatz, Beerenstrasse 50, 14163 Berlin, Germany; ^4^Department of Clinical Chemistry, St. Antonius Hospital, Koekoekslaan 1, 3435 CM Nieuwegein/Utrecht, Netherlands

In the article titled “Expression Analysis of Fibronectin Type III Domain-Containing (FNDC) Genes in Inflammatory Bowel Disease and Colorectal Cancer” [1], there were errors in Figures 2 and 3 and Section 3.5. The corrected figures are shown below and are listed as Figures 2 and 3. Also, the incorrect value in Section 3.5 is corrected below.

## 3.5 FNDC and GPR116 Expression in GEO Data Sets

Publicly available data sets of the GEO database were analyzed to complement our findings with previous comparable studies. First, the microarray expression of FNDCs in seven human colonic tumor cell lines from the NCI-60 panel was obtained (GDS4296) [30]. All FNDCs were expressed, whereas FNDC3A displayed the highest expression values (mean 2.4-fold higher) in all cell lines (Figure 4(a)). Significant expression differences (*p* < 0.05) in analyzed cell lines were found regarding FNDC3A and FNDC3B (significance bars are not shown for overview purposes). In the microsatellite-unstable colorectal cancer data set (GDS4515) of human MSI CRC samples (*n* = 34), we analyzed the gene expressions compared to nonaffected colonic mucosa (*n* = 15) [31]. The expression data of FNDC3A, FNDC3B, FNDC4, and GPR116 were available and showed a small but significant downregulation of FNDC4 in MSI CRCs (*p* = 0.047, Figure 4(b)), while FNDC3A (*p* = 0.055) and FNDC3B (*p* = 0.127) remained unchanged. GPR116 was significantly upregulated (*p* = 0.014). To test for possible confounding by age or sex, we analyzed expression data in early- and late-onset CRCs in females (*n* = 21) and males (*n* = 25), diagnosed at an age of 28-53 years (*n* = 27) or 69-87 years (*n* = 19) (GDS5232) [32]. Data were available for FNDC3A, FNDC4, and GPR116, which showed neither age- (*p* = 0.548, *p* = 0.906, and *p* = 0.160) nor sex-dependent differences (*p* = 0.438, *p* = 0.629, and *p* = 0.547).

## Figures and Tables

**Figure 1:**
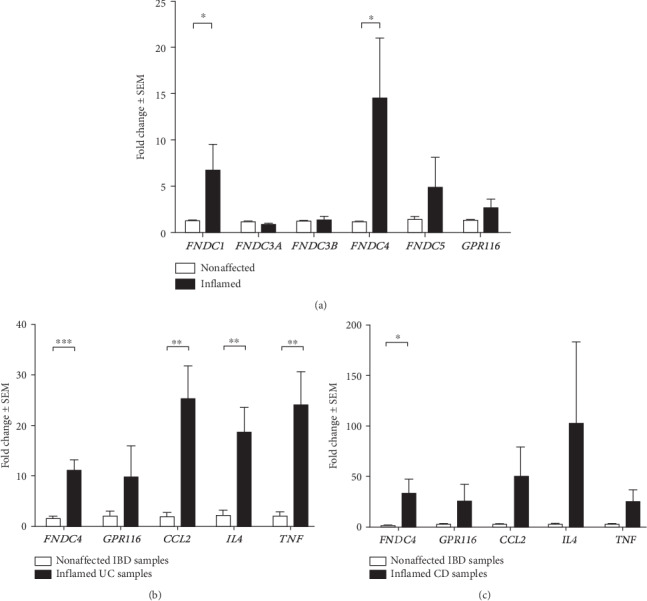
FNDC and GPR116 expression in nonaffected vs. inflamed mucosal samples of IBD patients. Expression levels of FNDC1 and FNDC4 were significantly higher in inflamed samples (*n* = 14) than in nonaffected samples (*n* = 22) of IBD patients (a). FNDC4, GPR116, CCL2, IL-4, and TNF expression levels in samples of active ulcerative colitis (UC, *n* = 8) (b) or Crohn's disease (CD, *n* = 8) (c), as compared to nonaffected samples. ^∗^*p* < 0 05, ^∗∗^*p* < 0 01, and ^∗∗∗^*p* < 0 001.

**Figure 2:**
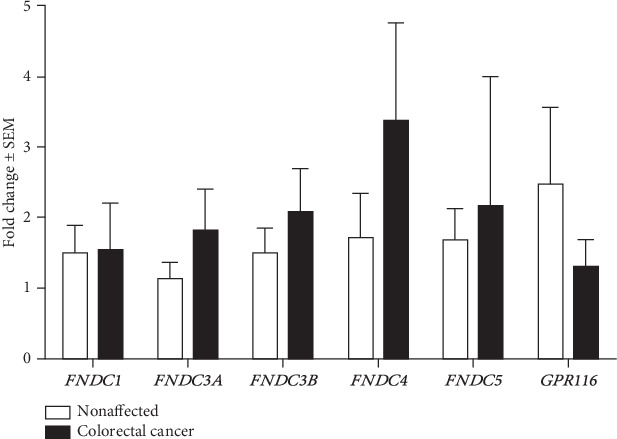
FNDC and GPR116 expression in colorectal cancer. No significant expression differences were found in a paired-sample *t*-test analysis for any of the investigated genes between cancerous samples and the surrounding nonaffected samples (*n* = 8-10).
