# Supplementary material for: Expression Analysis of Fibronectin Type III Domain-Containing (FNDC) Genes in Inflammatory Bowel Disease and Colorectal Cancer
Source: Gastroenterol Res Pract. 2019 Apr 9;2019:3784172. doi: 10.1155/2019/3784172 (PMC6481110; doi:10.1155/2019/3784172)
Supplement: Supplementary Materials — Supplemental figure: immunofluorescence staining and autofluorescence of inflamed intestinal tissue. (a) A subepithelial cellular IF label is visible with the use of 1st antibody: Anti-FNDC4 (HPA015804, dilution 1 : 50, Sigma-Aldrich) and 2nd antibody: Goat Anti-Rabbit IgG H&L (Alexa Fluor® 594) (ab150080, dilution 1 : 200, Abcam). Colonic crypts are labeled by Cadherin-17 antibody (MAB1032, R&D Systems) with 2nd antibody Goat Anti-Mouse IgG FITC (F0257, dilution 1 : 250, Sigma-Aldrich). DAPI was used for counterstaining. (b) The subepithelial cellular label also appears in a labeling control without antibodies after an excitation with 594 nm and is therefore autofluorescence. [file 3784172.f1.pdf]

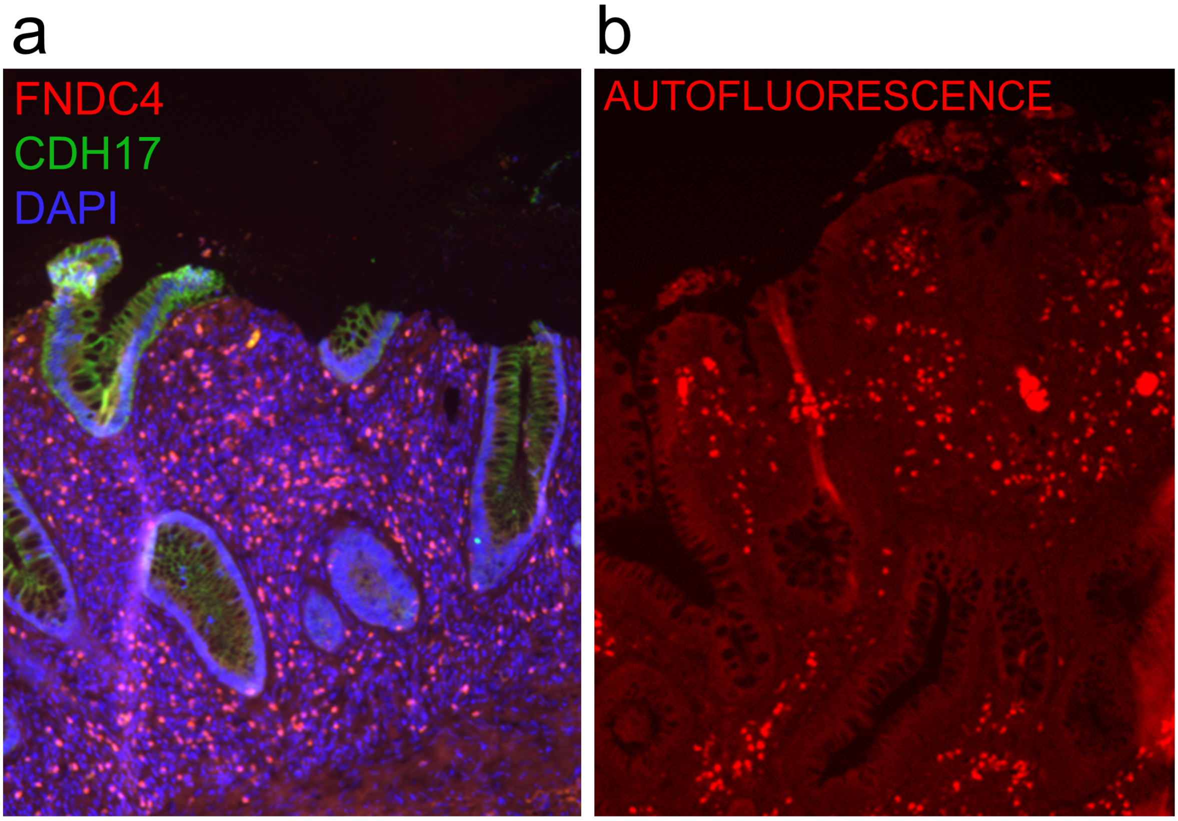

Supplemental figure: Immunofluorescence staining and autofluorescence of inflamed intestinal tissue. a) a subepithelial cellular IF label is visible with the use of 1st antibody: Anti-FNDC4 (HPA015804, dilution 1:50, Sigma-Aldrich) and 2nd antibody: Goat Anti-Rabbit IgG H&L (Alexa Fluor® 594) (ab150080, dilution 1:200, Abcam). Colonic crypts are labeled by Cadherin-17 antibody (MAB1032, R&D Systems) with 2nd antibody Goat Anti-Mouse IgG FITC (F0257, dilution 1:250, Sigma-Aldrich). DAPI was used for counterstaining. b) the subepithelial cellular label also appears in a labeling control without antibodies after an excitation with 594nm and is therefore autofluorescence.
